# Canada

**Published:** 1897-01

**Authors:** 


*Canada.* Overtures are in progress between representatives of Canada and the United States reciprocally to release the quarantine measures now existing relative to cattle.

---

Glutol continues to be highly commended by German veterinarians in the treatment of wounds as a dressing-powder. Harness- and saddle-galls, fistulous tracts, irritating eczemas, scratches, lacerated wounds, and the treatment of a number of cows whose calves had been dying regularly from diarrhœa shortly after birth, are among the cases reported where very favorable results were obtained. In this last condition glutol tampons were placed deep in the vaginas of the cows five days before calving and the external parts washed with a 3 per cent. solution of creolin.

Equal parts of a 1 per cent. aqueous solution of trikresol and hot water, applied with a camel's-hair brush, quickly eradicated an extremely irritating, inflammatory eruptive skin affection of a canary-bird, attacking both eyelids, the throat, butt of one wing, and one leg, at the Philadelphia Veterinary Sanitarium.

Useful formula for rheumatoid influenza:

℞.—Sodii salicylas, ʒiij; potass. iodidii, ʒiss; pulv. glychr. rad. and pulv. fenugræci, āā ʒss. M.—Ft. chartæ No. xvi.

Sig.—One powder every four hours, as required. Externally to local parts apply soap liniment, four portions; tinctures of aconite, opium, belladonna, and iodine, of each one portion.

